# Pentagalloyl Glucose- and Ethyl Gallate-Rich Extract from Maprang Seeds Induce Apoptosis in MCF-7 Breast Cancer Cells through Mitochondria-Mediated Pathway

**DOI:** 10.1155/2020/5686029

**Published:** 2020-04-21

**Authors:** Jiraporn Kantapan, Siwaphon Paksee, Pornthip Chawapun, Padchanee Sangthong, Nathupakorn Dechsupa

**Affiliations:** ^1^Department of Radiologic Technology, Faculty of Associated Medical Sciences, Chiang Mai University, Chiang Mai 50200, Thailand; ^2^Department of Radiological Technology, Kanchanabhishek Institute of Medical and Public Health Technology, Nonthaburi 11150, Thailand; ^3^Interdisciplinary Program in Biotechnology, Graduate School, Chiang Mai University, Chiang Mai 50200, Thailand; ^4^Department of Chemistry, Faculty of Science, Chiang Mai University, Chiang Mai 50200, Thailand; ^5^Research Center on Chemistry for Development of Health Promoting Products from Northern Resources, Faculty of Science, Chiang Mai University, Chiang Mai 50200, Thailand

## Abstract

*Bouea macrophylla* Griffith, locally known as maprang, has important economic value as a Thai fruit tree. The maprang seed extract (MPSE) has been shown to exhibit antibacterial and anticancer activities. However, the bioactive constituents in MPSE and the molecular mechanisms underlying these anticancer activities remain poorly understood. This study aims to identify the active compounds in MPSE and to investigate the mechanisms involved in MPSE-induced apoptosis in MCF-7 treated cancer cells. The cytotoxic effect was determined by MTT assay. The apoptosis induction of MPSE was evaluated in terms of ROS production, mitochondrial membrane potential depolarization, and apoptosis-related gene expression. The compounds identified by HPLC and LC/MS analysis were pentagalloyl glucose, ethyl gallate, and gallic acid. MPSE treatment decreased cell proliferation in MCF-7 cells, and MPSE was postulated to induce G2/M phase cell cycle arrest. MPSE was found to promote intracellular ROS production in MCF-7 treated cells and to also influence the depolarization of mitochondrial membrane potential. In addition, MPSE treatment can lead to increase in the *Bax/Bcl-2* gene expression ratio, suggesting that MPSE-induced apoptosis is mitochondria-dependent pathway. Our results suggest that natural products obtained from maprang seeds have the potential to target the apoptosis pathway in breast cancer treatments.

## 1. Introduction

Breast cancer is the leading form of cancer among women globally and stands out as a significant influencer of their morbidity and mortality rates [1]. Conventional therapy for breast cancer, including those that involve surgical procedures, chemotherapy, and radiotherapy have been improved in recent years to enhance treatment efficacy and reduce the number of cancer-related deaths among women. However, continuous use of chemotherapeutic agents or radiation against breast cancer has frequently contributed to the problem of therapy resistance. The underlying mechanism involved in conventional therapies is the activation of the antiapoptosis pathway [2, 3]. Resistance to apoptotic cell death in cancer cells represents one of the major obstacles to eliminating cancer cells. Current research efforts have been focused on the identification of certain compounds that are able to effectively trigger apoptosis. Moreover, an ideal anticancer drug must be selective and cytotoxic to cancer cells without resulting in adverse effects on normal cells [4]. Apoptosis, a type of programmed cell death, is commonly considered a prevalent form of cell death [5]. The underlying mechanism of apoptosis occurs through the mitochondria-dependent or mitochondria-independent pathway [6]. The mitochondria-dependent pathway (intrinsic pathway) is mainly triggered by nonreceptor stimuli including DNA damage and oxidative stress [7, 8]. Reactive oxygen species (ROS) play a crucial role in cellular function and cancer progression. Mitochondria are a major source of cellular ROS and the excessive generation of ROS, which can lead to mitochondrial dysfunction and thereby induction of apoptotic cell death [9]. It is well known that cancer cells display the distinct feature of high oxidative stress, which in turn exposes these cancer cells and makes them more vulnerable to further oxidative stress [10]. Therefore, targeting ROS holds great promise and may be an important aspect of an effective method of cancer treatment.

Plant-derived phytochemicals have been suggested as potential anticancer agents due to their low toxicity to normal cells and their high efficacy. In fact, most of the clinically applied anticancer drugs are produced from plants such as etoposide, topotecan, vinblastine, and vincristine [11]. Recently, numerous natural products were found to possess a cytotoxic effect by inducing apoptosis in cancer cells. These substances can also be used in combination with chemotherapy or radiotherapy, which can enhance the therapeutic efficacy and reduce side effects of many common cancer treatments [12, 13]. Many researchers are now paying attention to investigations on the potential of plants that can produce phytochemical compounds that can become useful to the pharmaceutical industry. Particularly, 1,2,3,4,6-penta-O-galloyl-*β*-D-glucose or pentagalloyl glucose (PGG) is a hydrolysable tannin that belongs to a group of gallotannins and is a naturally occurring polyphenol compound found in various medicinal herbs and plants such as *Galla Chinensis*, *Galla Rhois*, and *Paeonia lactiflora* [14]. PGG has attracted attention because of its therapeutic potential and has shown certain functional properties such as antimicrobial, anti-inflammatory, anticancer, antidiabetic, and antioxidant activities [15]. PGG possesses antiproliferative effects on a variety of cancer cells including prostate cancer [16], liver cancer [17], and breast cancer [18].

Although PGG has been identified in plants that are commonly used in Chinese medicine, recent researchers have identified PGG in a number of agroindustrial by-products such as mango seed kernels and the seeds of *Oenothera paradoxa* [19, 20]. Food waste and by-products are recognized as new and cheap sources of valuable components that have garnered greater amounts of attention. In recent years, there has been increased interest in the possibility of obtaining added value from agroindustrial waste [21]. It has been well-established that many plant by-products (peels, pulps, and seeds) are valuable sources of nutrients and contain a variety of bioactive molecules [22, 23]. The recovery and utilization of valuable compounds obtained from plant by-products would have a significantly positive impact on the potential socioeconomic benefits in relevant plant-producing areas.

Marian plums (*Bouea macrophylla* Griffith) are native fruits to Southeast Asia and are known as “maprang” in Thailand. The species belong to the same family as mangos (Anacardiaceae). Maprang trees are popular and important economic fruit trees in Thailand. Generally, maprang fruits are either consumed fresh or are processed for use in a range of products such as juices, desserts, and pickled snacks. Apart from the pulp that is routinely consumed, the seed is usually removed by the consumer as a form of waste. These noncommercial by-products may be an exploitable source of natural ingredients in various applications. By-products such as these can be used as phytochemical pharmaceutical substances in the prevention or treatment of a number of human diseases. It has been reported that the maprang seed extract or MPSE exhibits antiproliferative activities against drug-sensitive and drug-resistant leukemic and lung cancer cells [24]. Recently, we have reported that the HPLC profiling of the hydroethanolic extract of maprang seeds (MPSE) contains four major compounds. MPSE is known to exert antioxidant and broad spectrum antibacterial activities and to display anticancer effects on tumor cells [25]. Interestingly, the pretreatment of MCF7 cells with MPSE, before being exposed to X-ray radiation, prohibits the epithelial-mesenchymal transition (EMT) process and reduces the onset of cancer stem cells (CSCs) that are generated in the posttreatment phase in response to stressful stimuli. Moreover, the pretreatment of MPSE suppresses the expression of multidrug resistance proteins, which are involved in the therapeutic resistance process [26, 27]. These all suggest that MPSE could be an important component of an effective form of therapy for cancer. However, the underlying mechanisms of the suppression of cancer proliferation have not yet been fully investigated or reported on.

Herein, we aimed to investigate the anticancer effects of MPSE on the human breast cancer MCF-7 cell line model and to explore the underlying mechanisms that are involved. Liquid chromatography-mass spectrometry (LC-MS) and high-performance liquid chromatography (HPLC) profile were utilized to explore the potential phytochemicals responsible for the relevant anticancer activities. In order to explore the possible mechanisms of MPSE, the effect of MPSE on ROS generation, the cell cycle progression, the disruption of mitochondrial membrane potential, the altered expression of *Bax* and *Bcl-2* genes, and the process of apoptosis induction were all investigated in MCF-7 cells. The findings may lead to the establishment of a new alternative drug that could improve the curative effects of effective breast cancer treatments.

## 2. Materials and Methods

### 2.1. Materials and Reagents

Acetonitrile, trifluoroacetic acid (TFA), ethanol, and methanol were obtained from Sigma-Aldrich (St. Louis, USA). HPLC-grade acetonitrile and DMSO were obtained from Merck (Darmstadt, Germany). RPMI 1640, Dulbecco's Modified Eagle's Medium/Nutrient Mixture F-12 (DMEM/F-12), Trypsin-EDTA, streptomycin, and penicillin were purchased from Caisson Lab (Utah, USA). Fetal bovine serum was supplied by Sigma-Aldrich (St. Louis, USA). Additionally, 3-(4,5-dimethylthiazol-2-yl)-2,5-diphenyltetrazolium bromide (MTT), human insulin, epidermal growth factor, and hydrocortisone were purchased from Sigma (St. Louis, USA). Annexin-V-FITC Apoptosis Detection Kit and dichlorodihydrofluorescein diacetate (DCFH-DA) were also purchased from Sigma. JC-1 Mitochondrial Membrane Potential Assay Kit was procured from Calbiochem (California, USA). Additionally, 4′,6-diamidino-2-phenylindole dihydrochloride (DAPI) was obtained from Thermo Fisher Scientific (MA, USA). All standard compounds including gallic acid (GA), ethyl gallate (EG), and penta-O-galloyl-*β*-D-glucose hydrate (PGG) were purchased from Sigma-Aldrich (St. Louis, USA).

### 2.2. Plant Materials and Extraction

Fifty kilograms of maprang fruit, both unripe (more than 50 days after anthesis), and ripened specimens of two varieties, including maprang wan (sweet maprang) and maprang prieyo (sour maprang), were harvested during the period of March–April 2017 from the Marian Plum Plantation located in Nakhon Nayok Province, Thailand. The fruit was triple-washed with tap water and transverse-sectioned. Seeds were removed, weighed, and minced. MPSE was extracted according to the method described by Dechsupa et al. [25]. Briefly, minced seeds were dried with hot air at 60°C for further extraction. Subsequently, 350 g of minced seeds was immersed in 3.5 L of hydroethanolic systems (75%EtOH) in order to macerate them for 7 days with daily shaking. The extraction solutions were filtered through Kieselguhr and were dried by evaporation at 40°C at an approximate rotation speed of 200 rpm using a rotary evaporator (Buchi Rotavapor R-210, Switzerland). The crude extract of the maprang seeds was collected and stored at room temperature in a desiccator for further study. To prepare the stock solution, the powder samples of MPSE were solubilized with DI water at a final concentration of 1 mg/mL. The solution was then filtered with a 0.22 *μ*m syringe filter, aliquoted, and stored at −70°C until being used.

### 2.3. HPLC Analysis of PGG, EG, and GA in MPSE

HPLC analysis of MPSE was characterized and identified by reverse phase HPLC (LC-20AD Prominence Liquid Chromatograph equipped with SPD-M20A Prominence UV-Vis Diode Array Detector; Shimadzu, Japan). The analytical column used involved 250 mm × 4.6 mm i.d., 5 *μ*m ZORBAX Eclipse Plus-C18 with a guard column of 12.5 mm × 4.6 mm i.d., 5 *μ*m ZORBAX Eclipse Plus-C18 (Agilent, USA) and was operated at 25°C. The mobile phase consisted of 0.1 (v/v) trifluoroacetic acid (TFA) in water (eluent A) and acetonitrile (eluent B). The flow rate was recorded at 1 mL/min and the linear gradient program was optimized by specific indications of time (min) and eluent B (%) as follows: 0, 5; 5, 5; 7, 10; 12, 10; 12, 10; 14, 15; 19, 15; 21, 20; 26, 20; 30, 25; 35, 25; 37, 30; 45, 30; 50, 100; 55, 100; 60, 5; and 65, 5. Stock solution of maprang seed extract (MPSE) was freshly prepared at 2000 *μ*g/mL with Type I water before being used and was sterilized through the use of a 0.2 *μ*m syringe filter under the biosafety cabinet for further study. The stock solution of gallic acid (GA; 6.25–250 *μ*g/mL) and pentagalloyl glucose (PGG; 6.25–400 *μ*g/mL) was serially diluted with water, whereas ethyl gallate (EG; 25–500 *μ*g/mL) was diluted with DMSO to obtain seventh concentration levels for each analyte in order to prepare a calibration curve. Method validation was performed in accordance with the ICH guidelines [28]. The linearity was assessed by calculating the coefficient of determination (*r*^2^), which should be more than 0.999. The quantification of analyte was accomplished by measuring the area peak. The limit of detection (LOD) and the limit of quantification (LOQ) were determined by the signal-to-noise ratio obtained from the calibration curve based on the standard deviation (SD) of the response on the slope. Three major phytochemicals of MPSE were identified by spiking a standard solution of GA, EG, and PGG into 500 *μ*g/mL MPSE solution with final concentrations of 10, 100, and 37.5 *μ*g/mL, respectively. The quantities of GA, EG, and PGG in 1000 *μ*g/mL MPSE were measured from an area peak of 20 *μ*L injected volume of each analyst in triplicate.

### 2.4. LC-MS Analysis of PGG, EG, and GA in MPSE

LC-MS analysis of gallotannin profiles of maprang seed extract (10 *μ*L injected volume of 500 *μ*g/mL MPSE) was characterized by HPLC using a RP-C18 LiChroCART RP-18e column (125 mm × 4.6 mm, 5 *μ*m) (Merck, USA). The eluents were 0.1% TFA in water (A) and 100% HPLC-grade acetonitrile (ACN) (B) (Merck). Chromatographic conditions (time (min), B (%)) were as follows: 0, 5; 5, 5; 7, 10; 12, 10; 12, 10; 14, 15; 19, 15; 21, 20; 26, 20; 30, 25; 35, 25; 37, 30; 45, 30; 50, 100; 55, 100; 60, 5; and 65, 5. The flow rate was 1 mL/min. Detection was performed with a UV detection system L-7400 LaChrom Merck Hitachi (Merck KGaA) at 270 nm and a Hewlett Packard 1100 Series Diode Array Detector (DAD) (Agilent Technologies, Germany). Mass spectra systems were registered by a Hewlett Packard 1100 MSD SL (Agilent Technologies, USA), operating in nitrogen flow at atmospheric pressure, and by applying the electrical ionization (API-ES) mode. The voltage in the capillary was 4000 V (positive) and 3500 V (negative). The flow rate of nitrogen was 13 L/min, and the temperature was set at 320°C. The scanning range was set at 70–1000 m/z (positive) with an interval of 0.20 m/z.

### 2.5. Cell Lines and Culture Conditions

Estrogen receptor-positive breast cancer cell MCF-7 (ATCC®HTB-22™), triple-negative breast cancer cell MDA-MB-231 (ATCC®HTB-26™), and normal mammary epithelial MCF-10A (ATCC®CRL-10317™) cell line were purchased from the American Type and Culture Collection (Manassas, VA, USA). MCF-10A was maintained in DMEM/F12 supplemented with 10% fetal bovine serum (FBS), 2 mM glutamine, 0.5 *μ*g/mL hydrocortisone, 10 *μ*g/mL insulin, 20 ng/mL human epidermal growth factor, and 1% penicillin-streptomycin. MCF-7 and MDA-MB-231 cells were maintained with RPMI 1640 medium supplemented with 10% fetal bovine serum (FBS) and 1% penicillin-streptomycin in a 95% air humidified atmosphere with 5% CO_2_ at 37°C.

### 2.6. Cell Viability by MTT Assay

Cellular proliferation and the inhibitory effect of MPSE were determined by MTT (3-(4,5-dimethylthiazol-2-yl)-2,5-diphenyltetrazolium bromide) assay [26]. Briefly, cells were seeded into a 24-well plate and cultured for 24 h before being treated with the various concentrations of MPSE (0–90 *μ*g/mL). After incubation with MPSE for 24, 48, and 72 h, a 1 mg/mL final concentration of MTT solution was added and cells were further incubated for 4 h to allow for the forming of formazan crystals. After incubation, the solution in each well was discarded and 200 *μ*L of DMSO was added to solubilize the formazan crystals. The absorbance of the formazan solution was measured at 560 nm by UV-Vis spectrophotometer (Agilent 8453, USA). The graph of percentage of cell viability versus concentrations of MPSE was plotted, and the cytotoxicity effect was determined by IC_50_, which refers to the concentration of MPSE required for inhibiting 50% of cell growth when compared to the controls.

### 2.7. Cell Cycle Analysis by Flow Cytometry

Changes in cell cycle distribution induced by MPSE were analyzed using propidium iodide (PI) staining. In brief, cells were seeded into 6-well plates and treated with various concentrations of MPSE for 24 and 48 h. After completion of the incubation phase, both the suspension cells and the adherent cells were harvested, washed with PBS, and centrifuged at 7,000 rpm for 1 min. The supernatant was discarded, and the pellets were collected and then fixed with 70% ethanol overnight at 4°C. Subsequently, the fixed cells were washed with ice-cold PBS before being stained with the binding buffer containing 0.1% Triton X-100, 0.2 mg/mL of RNase, and 10 *μ*g/mL of PI. Stained cells were then incubated at 37°C for 30 minutes in the dark. Finally, the stained cells were analyzed by flow cytometry (Beckman Coulter, Epics XL-MCL). Flow cytometric data were then analyzed by FlowJo 10 software.

### 2.8. Determination of Intracellular ROS Levels in MPSE-Treated MCF-7 Cells

The intracellular ROS levels were determined using dichlorodihydrofluorescein diacetate (DCFH-DA) assay. In brief, 2 × 10^5^ cells were seeded in 6-well plates and cultured for 24 h to allow cell attachment to occur. Cells were treated with various concentrations of MPSE for 3, 6, 9, 12, and 24 h. After that, cells were harvested by trypsinization and then centrifuged at 7,000 rpm for 1 min. The supernatant was discarded, and cell pellets were collected. Cell pellets were then washed with ice-cold PBS, incubated with 1 *μ*M of DCFH-DA for 30 min at 37°C, and kept away from light. Subsequently, cells were washed again with PBS and then immediately placed on ice before performing flow cytometer analysis. Flow cytometric data were analyzed using FlowJo 10 software.

### 2.9. Mitochondrial Membrane Potential (ΔΨm) Analysis by Flow Cytometry

The loss of ΔΨm was evaluated by using flow cytometry. Cells were induced to encounter apoptosis by being incubated with various concentrations of MPSE for 12 and 24 h, while the negative control was incubated in the absence of MPSE. Cells were harvested and centrifuged at 7,000 rpm for 1 min. The supernatant was discarded, and cell pellets were resuspended again in 0.5 mL PBS containing JC-1 dye solution at a final concentration of 2.5 *μ*g/mL. Cell pellets were then incubated at 37°C for 15 min. Samples were washed again with 0.5 mL warm PBS before performing flow cytometer analysis using an excitation wavelength of 488 nm. The obtained data were analyzed by JC-1 monomer and were further detected in the FL1 channel and JC-1 aggregates in the FL2 channel.

### 2.10. Morphological Detection of Apoptosis by DAPI Staining

Morphological characteristics of apoptotic cells were determined using 4′,6-diamidino-2-phenylindole dihydrochloride (DAPI) staining. In brief, 2 × 10^5^ cells were seeded in 6-well plates and cultured for 24 h to allow cell attachment to occur. Cells were treated with various concentrations of MPSE for 24 h. After treatment with MPSE, the cells were washed with PBS, then fixed with 4% formaldehyde in PBS for 15 min at room temperature, and then washed twice with PBS. Cells were permeabilized with permeabilization buffer containing 1% BSA and 0.2% Triton X-100 in PBS for 1 h before being incubating with 300 nM DAPI staining solution for 5 min. Cells were protected from light throughout the procedure. The cells were washed twice with PBS and photographed with a fluorescence microscope (Nikon, Eclipse Ts2).

### 2.11. Apoptosis Determination Using Annexin-V FITC/PI Staining by Flow Cytometry

Annexin-V FITC/PI staining was used to detect apoptotic cells. Cellular populations were easily distinguished by Annexin-V FITC-bound cells and were considered an early sign of apoptosis, while PI-bound cells served as indicators of necrosis. Additionally, both dye-bound cells (Annexin-V FITC/PI) served as indicators of late apoptosis/necrosis. Briefly, cells were seeded at a density of 2 × 10^5^ cells in 6-well plates and cultured for 24 h to allow cell attachment to occur. Cells were treated with various concentrations of MPSE and then incubated for 24 h. Cells were harvested by trypsinization and centrifuged at 7,000 rpm for 1 min. The supernatant was discarded, and cell pellets were washed with PBS before being stained with Annexin-V FITC/PI. The cell pellets were then resuspended in 100 *μ*L of 1X binding buffer and 5 *μ*L of Annexin-V FITC, and 10 *μ*L of PI solution was added until the vortex was completely mixed. The mixture was incubated at room temperature for 15 min and kept away from light. Finally, 400 *μ*L of 1X binding buffer was added, and the samples were immediately analyzed by flow cytometry (Beckman Coulter, Epics XL-MCL). Each sample required the presence of 10,000 cells for the data to be collected. Flow cytometric data were analyzed using FlowJo 10 software. Fluorescence distribution was displayed as a percentage of fluorescent cells in each quadrant.

### 2.12. Apoptosis-Related Gene Expression by RT-PCR (Reverse Transcription Polymerase Chain Reaction)

Total RNA was extracted using an E.Z.N.A.® Total RNA Kit I (Omega Bio-tek, USA), and reverse transcription reactions were performed using the ReverTra Ace® qPCR RT Master Mix with a gDNA Remover Kit (TOYOBO, Japan) according to the manufacturer's guidelines. Additionally, 10 *μ*L of reaction mixture was obtained by PCR with 30 cycles. PCR products were resolved in 1.5% agarose gel that was stained with RedSafe™ nucleic acid staining solution. *GAPDH* (glyceraldehyde 3-phosphate dehydrogenase) was used as a loading control, which was considered as a housekeeping gene. The primer sequences are listed below.

*Bax* forward primer: 5ʹ- CCC CCG AGA GGT CTT TTT CC- 3ʹ

Reverse primer: 5ʹ- GGA CAT CAG TCG CTT CAG TG- 3ʹ

*Bcl-2* forward primer: 5ʹ- GAA CTG GGG GAG GAT TGT GG- 3ʹ

Reverse primer: 5ʹ- TTC ACT TGT GGC CCA GAT AGG- 3ʹ

*PARP* forward primer: 5ʹ- GGC AAG CAC AGT GTC AAA GG- 3ʹ

Reverse primer: 5ʹ- GGC TAC CTC TCC CAA TTA CC- 3ʹ

*GAPDH* forward primer: 5ʹ- CACCATCTTCCAGGAGCGAGATC- 3ʹ

Reverse primer: 5ʹ- GTGGTGCAGGAGGCATTGCTGA- 3ʹ

### 2.13. Statistical Analysis

Data were expressed as mean ± standard deviation (SD) of at least three independent experiments. The data were analyzed by one-way analysis of variance (ANOVA), followed by Tukey's test to detect significant differences among means of each factor using OriginPro 2018 software. A value of *p* < 0.05 was considered statistically significant.

## 3. Results

### 3.1. Identification of 1,2,3,4,6-Pentagalloyl Glucose (PGG), Ethyl Gallate (EG), and Gallic Acid (GA) as Bioactive Compounds in MPSE by HPLC and LC-MS

**Figure 1:**
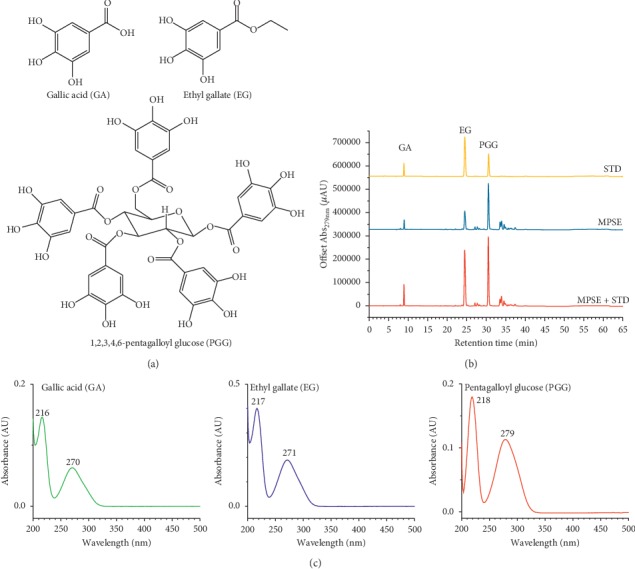
(a) Chemical structures of GA, EG, and PGG. (b) Identification of the three major phytochemicals, GA, EG, and PGG, in maprang seed extract (MPSE). (c) Absorption spectrum of GA, EG, and PGG. HPLC analysis was performed as described in the experimental section. HPLC: high-performance liquid chromatography. STD: standard.

The chemical structures of PGG, EG, and GA are shown in Figure 1(a). PGG, EG, and GA absorbed light energy at a maximum wavelength of 279 nm, 271 nm, and 270 nm, respectively, by HPLC analysis as shown in Figure 1(c). The absorption wavelengths at 271 nm and 279 nm were selected to monitor the quantity of each analyte, while the linear progression equations at indicated wavelengths of PGG, EG, and GA are listed in Table 1. The limit of detection (LOD) and limit of quantification (LOQ) (see Table 1) of PGG were similar to those of EG at 279 nm and were 0.09 and 0.28 *μ*g/mL, respectively. However, LOD and LOQ values at 271 nm of EG (LOD = 0.08 *μ*g/mL, LOQ = 0.26 *μ*g/mL) were slightly lower than those of PGG (LOD = 0.09 *μ*g/mL, LOQ = 0.28 *μ*g/mL), whereas GA revealed the same limits of detection at 0.11 *μ*g/mL for both wavelengths and was recorded at 271 nm and 279 nm. Additionally, the LOQ values of GA were 0.34 and 0.32 *μ*g/mL at wavelengths of 271 nm and 279 nm, respectively. In this study we proved that maprang seed extract (MPSE) is composed of PGG, EG, and GA as the major phytochemicals (Figure 1(b)). The retention time of GA, EG, and PGG that was monitored at 279 nm was found to be 9.00 ± 0.04, 24.35 ± 0.15, and 30.99 ± 0.23 min, respectively. The constituents of three phytochemicals in 1000 *μ*g/mL MPSE were determined using the calibration curves as indicated in Table 1. Using the area peaks at 271 nm, we obtained the concentrations of PGG, EG, and GA of 510.60 ± 47.86, 306.33 ± 21.06, and 26.83 ± 1.36 *μ*g/mL, respectively. For determination of the area peaks at 279 nm, we obtained the concentrations of PGG, EG, and GA at 155.46 ± 11.13, 91.72 ± 4.15, and 15.09 ± 0.98 *μ*g/mL, respectively. The summation of the concentration values of three phytochemicals was 843.10 ± 69.66 and 843.75 ± 70.28 *μ*g/mL for determination at 271 nm and 279 nm, respectively. The percentages of the three phytochemicals found in the maprang seed extract were 84.37 ± 8.33% (844 *μ*g/mL detected from 1000 *μ*g/mL and injected volume 20 *μ*L). Among the gallotannin content values, PGG revealed the highest content at 51.1% followed by EG (30.6%) and GA (2.7%).

**Figure 2:**
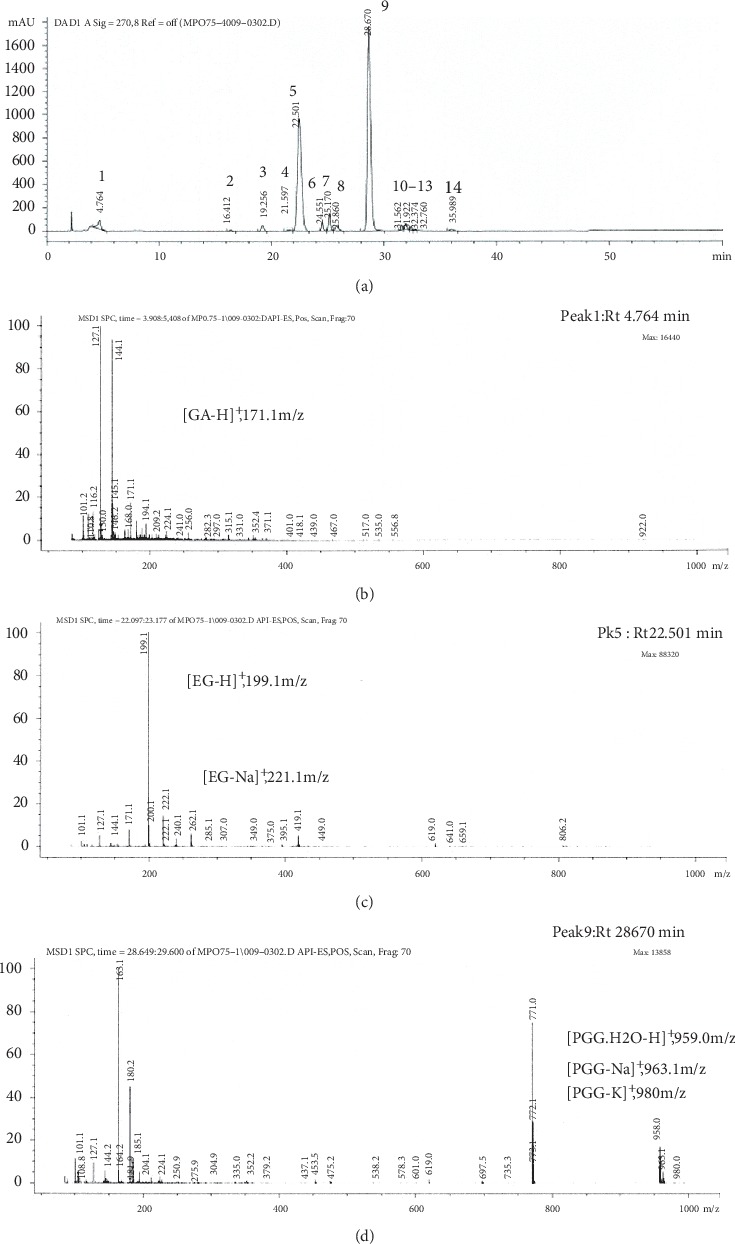
Phytochemical identification of MPSE. (a) LC chromatogram of maprang seed extract and mass spectrum of the three major phytochemicals as indicated by ^*∗*^: (b) peak no. 1 = GA; (c) peak no. 5 = EG; and (d) peak no. 9 = PGG.

PGG, EG, and GA were identified as the three major phytochemicals of maprang seed extract through HPLC analysis, as has been previously described. In this section, we confirmed the presence of these three compounds using LC-MS analysis with API-ES positive mode for determination of the molecular mass (m/z). The results are presented in Figure 2 and Table 2. According to the LC chromatogram of MPSE, as can be seen in Figure 2, a total of 14 compound peaks were observed. However, three of the fourteen compounds, peak no. 1 (Rt 4.764 min), peak no. 2 (Rt 22.501 min), and peak no. 9 (Rt 28.671 min), were identified for molecular mass. At Rt 4.764 min, the m/z ratio was recorded at 171.1 and was determined to be responsible for gallic acid (GA, molecular mass 170.12 Da) as [GA-H]^+^ and other adduct ions, [GA-Na]^+^, 194.1 m/z, that were observed. At Rt 22.501 min, ethyl gallate (EG, molecular mass 198.17 Da) corresponded with the results of two adduct ions as [EG-H]^+^, 199.1 m/z, and [GA-Na]^+^, 221.1 m/z, while at Rt 28.671 min, this compound corresponded with pentagalloyl glucose (PGG, molecular mass 940.68 Da). We found two forms of PGG, anhydrous and monohydrate, that were able to be adducted by Na^+^ ([PGG-Na]^+^, 963.1 m/z; [PGG.H_2_O-Na]^+^, 981.0 m/z) and H^+^ ([PGG.H_2_O-H]^+^, 959.0 m/z). Additionally, PGG was able to be adducted by K^+^ ([PGG-K]^+^, 980.0 m/z). The other fragments and adducted products associated with the API-ES positive mode of PGG, EG, and GA are presented in Table 2.

### 3.2. Antiproliferation Effect of MPSE on Human Breast Cancer Cells

**Figure 3:**
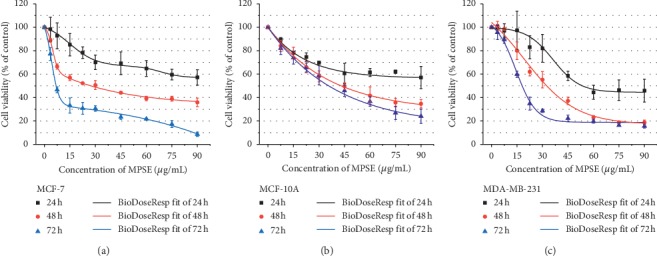
Effects of MPSE on cellular viability in human breast cancer MCF-7 and MDA-MB-231 cells. Cells were treated with different concentrations of MPSE (0–90 *μ*g/mL) for 24, 48, and 72 h. Then, the cell viability of the MCF-7 cells (a), the normal human breast cells MCF-10A (b), and the triple-negative breast cancer MDA-MB-231 cells (c) was assessed by MTT assay. Data are presented as mean ± SD of three independent experiments.

**Figure 4:**
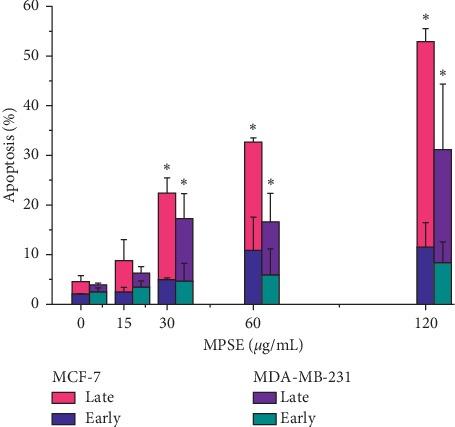
Effect of MPSE on apoptosis cell death at 48 h of treatment in MCF-7 and MDA-MB-231 cells. The percentage of apoptotic cells was analyzed by flow cytometry of Annexin-V/PI staining. Column graph representing quantification of apoptotic cells. Bars representing mean ± SD values of three independent experiments. ^*∗*^Statistically significant differences of total apoptosis from the control (*p* < 0.05).

The cytotoxicity effects of MPSE and isolated compounds were evaluated on MCF-7, MDA-MB-231, and MCF-10A cells using MTT assay. As shown in Table 3 and Figure 3, MPSE inhibited cellular proliferation of MCF7, MDA-MB-231, and MCF-10A cells in both a concentration-dependent manner (0–90 *μ*g/mL) and a time-dependent manner (24, 48, and 72 h). MPSE was the most cytotoxic towards MCF-7 cells, with IC_50_ value of 6.94 ± 1.57 *μ*g/mL at 72 h of incubation compared to MDA-MB-231 and MCF-10A with IC_50_ values of 18.92 ± 1.20 and 30.08 ± 3.70 *μ*g/mL, respectively. Considering MCF-7 cells, MPSE was the most cytotoxic compared to the isolated compounds, which suggested that the antiproliferative effect of MPSE against MCF-7 cells might be due to the synergistic effects of these bioactive compounds presented in the crude extract. PGG, which was the main bioactive component in MPSE, was least cytotoxic against MCF-7 and MCF-10A cells at 72 h (IC_50_ > 100 *μ*g/mL). However, the cytotoxic effect of PGG was more prominent on ER-negative MDA-MB-231 breast cancer cells with IC_50_ value of 26.46 ± 6.53 *μ*g/mL for 72 h of incubation. These results suggest that the cytotoxic effect of MPSE was more prominent on ER-positive MCF-7 breast cancer cells than on ER-negative MDA-MB-231 cells. This finding was supported by the percentage of apoptotic cell death determined by flow cytometry. According to the results, MPSE induced a higher percentage of apoptotic cell death against MCF-7 than MDA-MB-231 cells (Figure 4). Interestingly, the cytotoxic effect of MPSE was more prominent on breast cancer cells than on normal mammary epithelial MCF-10A cells. As can be seen, the doses required for MPSE to induce the growth of the inhibitory effects of MCF-7 were at least 4-fold lower than those of the MCF-10A cells. These results suggest that MPSE effectively induced the cytotoxic effects in breast cancer cells.

### 3.3. Effect of MPSE on Cell Cycle Distribution of Breast Cancer Cell MCF-7

**Figure 5:**
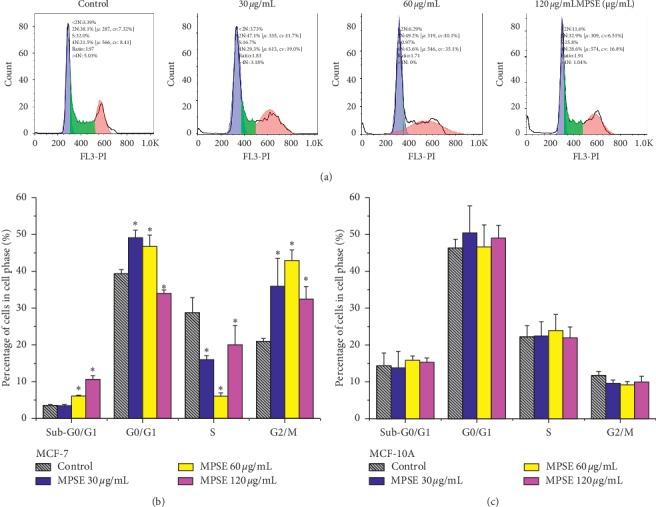
Cell cycle distribution of MCF-7 cells treated with MPSE at 24 h. Effects of MPSE on cell cycle distribution in MCF-7 and MCF-10A cells were analyzed using flow cytometry analysis of propidium iodide (PI) stained cells. (a) DNA histogram displayed cell cycle phase distribution of both the control and the MPSE-treated cells at 24 h. (b) Bar charts representing the percentage of cell populations in MCF-7 cells that were treated with MPSE. (c) Bar charts representing the percentage of cell populations in MCF-10A cells that were treated with MPSE. Data are presented as the mean ± SD of three independent experiments. ^*∗*^Statistically significant differences from the control (*p* < 0.05).

Cell cycle distribution of MCF-7 cells treated with MPSE at 24 h is shown in Figure 5. MCF-7 cells were treated with 30, 60, and 120 *μ*g/mL of MPSE for 24 h. MPSE treatment induced G2/M phase cell cycle arrest in a dose-dependent manner. The G2/M phase population increased significantly (*p* < 0.05) from 20.80% in the untreated cells to 30.57% and 40.20% in 30 and 60 *μ*g/mL of MPSE, respectively. Meanwhile, in 120 *μ*g/mL of MPSE, the cells continued cycling as the G0/G1, and G2/M phase populations were declined when compared to 60 *μ*g/mL of MPSE. This outcome was accompanied by an increase in the sub-G0/G1 and S phase populations. Moreover, the MPSE treatment of MCF-7 cells induced an increased accumulation of apoptotic sub-G0/G1 populations in a concentration-dependent manner. It should be noted that the MPSE treatment of the MCF-10A cell line did not reveal an influence of MPSE on cell cycle distribution (Figure 5(c)).

### 3.4. MPSE-Induced Intracellular ROS Generation and Triggered Disruption of Mitochondrial Membrane Potential (ΔΨm)

**Figure 6:**
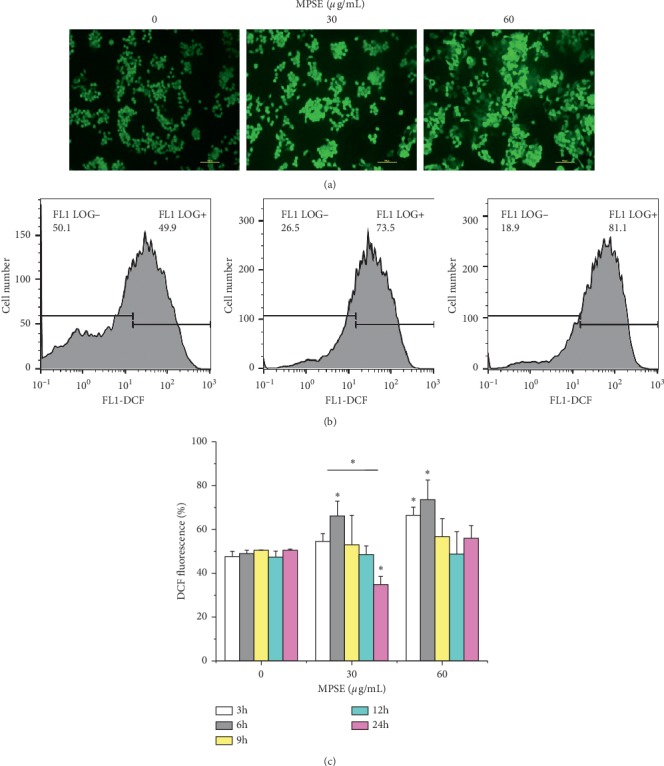
MPSE treatment induced intracellular ROS generation in MCF-7 cells. (a) Cells were treated with MPSE (0, 30, 60 *μ*g/mL) for 6 h, and generation of intracellular ROS was measured by DCHF-DA assay. (b) Time- and contribution-dependent changes of intracellular ROS by flow cytometry. Bars represent mean ± SD values of three independent experiments. ^*∗*^Statistically significant differences from the control (*p* < 0.05). ROS: reactive oxygen species.

**Figure 7:**
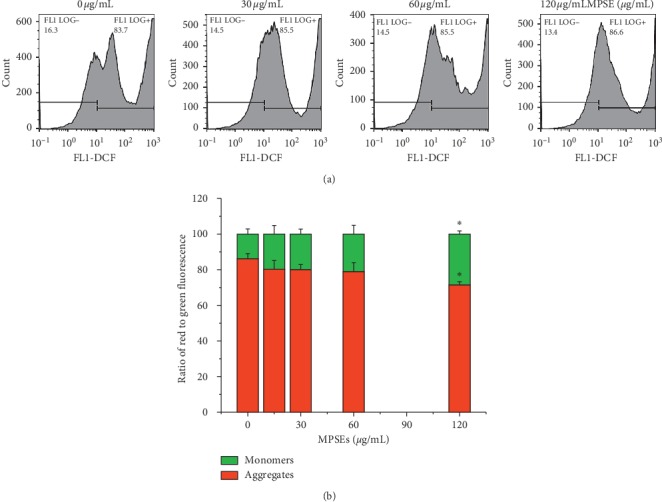
Study of MPSE treatment induced ROS generation and mitochondrial dysfunction in normal mammary epithelial MCF-10A cells. (a) Cells were treated with MPSE (0, 30, 60, 120 *μ*g/mL) for 24 h, and generation of intracellular ROS was measured by DCHF-DA assay. (b) The column graph represents the quantification of cells observed with green and red fluorescence (expressed in percentages). Cells were treated with MPSE (0, 15, 30, 60, 120 *μ*g/mL) for 24 h. Bars represent mean ± SD values of three independent experiments. ^*∗*^Statistically significant differences from the control (*p* < 0.05).

In order to determine the involvement of intracellular ROS in the cellular mechanism of the growth inhibitory effects of MPSE on MCF-7 cells, 2ʹ,7ʹ-dichlorofluorescin diacetate (H_2_DCFDA), the fluorescent probe, was used to measure intracellular ROS production induced by MPSE. With regard to MCF-7 cells that were exposed to 30 and 60 *μ*g/mL of MPSE for 6 h, the treatment with MPSE showed an increase in the proportion of cells with elevated green fluorescence intensity in a concentration-dependent manner (Figure 6(a)). In the treatment of MCF-7 cells with MPSE (30 and 60 *μ*g/mL) for 0–24 h, as is shown in Figure 6, MPSE provoked the increasing generation of intracellular ROS in a dose-dependent manner for 3–9 h. Subsequently, the values then progressively decreased with a significant (*p* < 0.05) reduction at 24 h. In contrast, MPSE had little impact on ROS generation in human normal breast epithelial MCF-10A cells, which correlated with mitochondrial membrane potential observed in MCF-10A (Figure 7). It was demonstrated that MPSE-induced ROS production resulted in partial mitochondrial membrane depolarization. These results explain the low cytotoxic effects of MPSE on normal breast epithelial MCF-10A cells.

**Figure 8:**
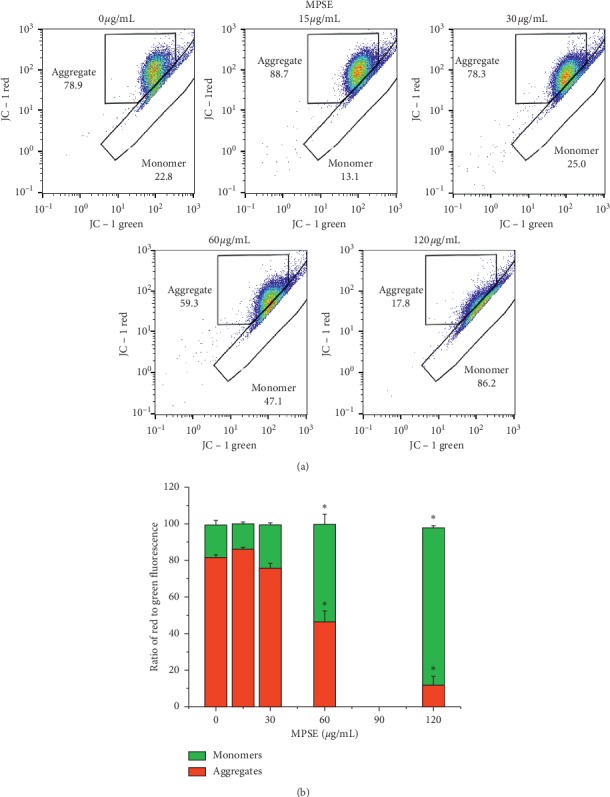
MPSE treatment induced mitochondrial dysfunction in MCF-7 cells as determined by JC-1 dye and flow cytometry analysis. (a) The dot plots of JC-1 represent red fluorescence against JC-1 green fluorescence, while the increment of green fluorescence indicates the loss of ΔΨm in the mitochondria of MPSE-treated MCF-7 cells. (b) The column graph represents the quantification of cells observed with green and red fluorescence (expressed in percentages).Bars represent mean ± SD values of three independent experiments. ^*∗*^Statistically significant differences from the control (*p* < 0.05).

Intracellular oxidative stress may result in a disruption of mitochondrial membrane potential (ΔΨm). Depletion of ΔΨm suggests that the mitochondrial membrane integrity was lost, which is reflected in the initial signal of apoptosis. To elucidate whether MPSE-induced ROS generation triggers mitochondria-mediated apoptosis, JC-1 fluorescent dye was used to assess the mitochondrial membrane permeabilization by flow cytometry at 12 and 24 h following treatment with MPSE (0–120 *μ*g/mL). Flow cytometry analysis of the fluorescent intensity ratio of red/green was determined (green fluorescence refers to JC-1 monomer at low membrane potentials, while red fluorescence refers to JC-1 aggregated at higher membrane potentials). Following MPSE treatments for 12 h, the treated MCF-7 cells showed an increase in the green fluorescent intensity due to the presence of monomeric JC-1 in a dose-dependent manner with 13.90%, 23.60%, 53.27%, and 85.93% for MPSE 15, 30, 60, and 120 *μ*g/mL, respectively. The untreated cells revealed 81.53% of red fluorescence and 17.80% of green fluorescence, which is indicative of cells with higher ΔΨm values (Figure 8). A similar trend was observed over 24 h of MPSEs treatment. It should be noted that MPSE gradually induced ROS generation over 3–9 h, with significant yields occurring at 6 h when the process was accompanied by concomitant disruption of ΔΨm at 12 h. This finding suggests that MPSE treatment induced excessive ROS generation leading to the disruption of ΔΨm and was involved in mitochondria-mediated apoptosis in MCF-7 cells.

### 3.5. MPSE-Induced Apoptosis in Breast Cancer Cell MCF-7

**Figure 9:**
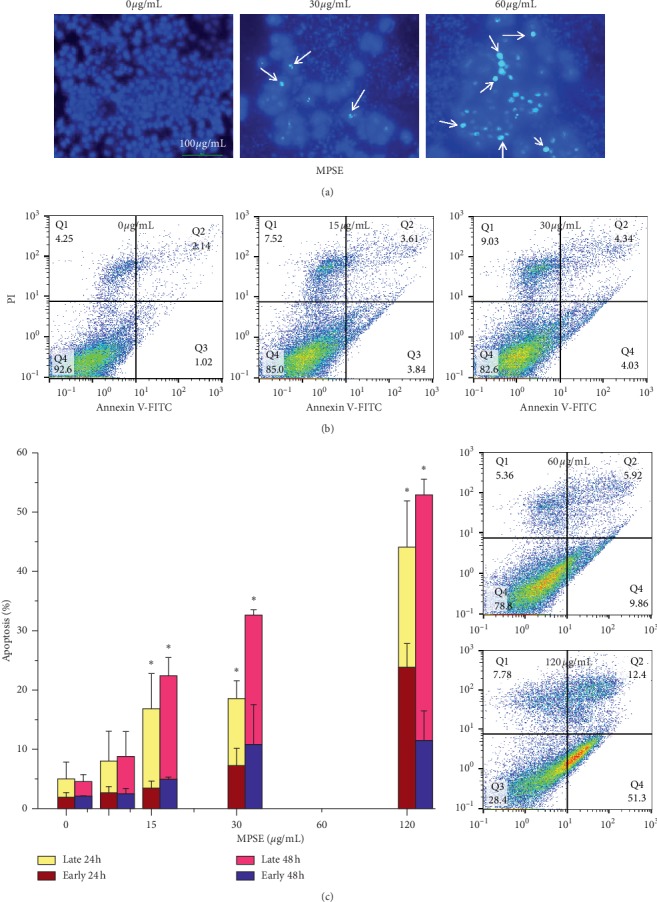
Induction of apoptosis in MCF-7 cells after MPSE treatment for 24 and 48 h (a) Determination of apoptosis in MCF-7 cells by DAPI staining. Cells were treated with MPSE at concentrations of 0, 15, 30, 60, and 120 *μ*g/mL for 24 and 48 h. Cells were then stained by DAPI and were observed under a fluorescence microscope. (b) The percentage of apoptotic cells was analyzed by flow cytometry of Annexin-V/PI staining. (c) Column graph representing quantification of apoptotic cells. Bars representing mean ± SD values of three independent experiments. ^*∗*^Statistically significant differences of total apoptosis from the control (*p* < 0.05). PI: propidium iodide.

To determine whether MPSE induces apoptotic cell death, the morphological characteristics of apoptotic cells were determined by staining the cells with DAPI (4′,6-diamidino-2-phenylindole dihydrochloride). MCF-7 cells that were treated with different concentrations of MPSE for 24 h revealed characteristic apoptotic morphology by emitting bright fluorescence, while fragment chromatin increased in a dose-dependent manner (Figure 9(a)). Additionally, apoptotic cell death was further quantified by Annexin-V FITC/PI double fluorescent staining. The externalization of phosphatidylserine, an indicator of the early stages of apoptosis, was analyzed by measuring Annexin-V binding. MCF-7 cells were treated with different concentrations (0–120 *μ*g/mL) of MPSE for 24 and 48 h. Percentages of cells undergoing apoptosis increased with increasing concentrations of MPSE and their treatment duration (Figures 9(b) and 9(c)). At 24 h of treatment, the percentage of apoptosis increased from 8.01 ± 1.03% to 44.11 ± 3.18% as observed in MCF-7 cells at 15 and 120 *μ*g/mL concentrations, whereas after 48 h of treatment, it was observed that much higher percentages, 8.8 ± 3.73% and 52.90 ± 2.65%, of cells underwent apoptosis at the same concentrations. Interestingly, we observed that after 24 h posttreatment the increase is mainly attributed to the promotion of early-stage apoptosis, while at 48 h the late-stage apoptosis was more prominent in MCF-7 treated with MPSE at the same concentration (Figure 9(c)). This result suggests that MCF-7 cells treated with MPSE-induced apoptotic cell death as the concentration of the treatment were increased over 24 h.

### 3.6. MPSE Increased *Bax/Bcl-2* Ratios in Breast Cancer Cells MCF-7

**Figure 10:**
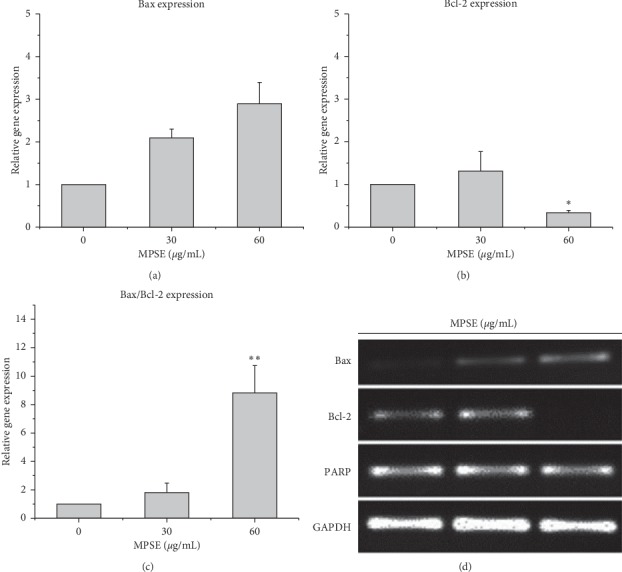
Expression of apoptosis-related genes in MCF-7 cells after MPSE treatment as determined by RT-PCR. MCF-7 cells were treated with MPSE at 30 and 60 *μ*g/mL for 48 h. The expression levels of *Bcl-2* family genes and the corresponding ratios of *Bax/Bcl-2* were assayed. MPSE upregulated the expression of *Bax* and downregulated the expression of *Bcl-2.* The expression of the genes was normalized against GAPDH and then compared to the control. The data are presented as the relative expression value of genes in the columns shown as ± SD of at least three replicates of three independent experiments. ^*∗*^Statistically significant differences from the control, *p* < 0.05;^*∗∗*^*p* < 0.01.

According to the apoptosis pathway, proapoptotic Bax and antiapoptotic Bcl-2 play important roles in apoptosis regulation as promoters or inhibitors of cell death. To evaluate the effects of MPSE on the expression of the apoptotic cell death pathway as related to the genes, the expressions of *Bax*, *Bcl-2*, and *PARP* were determined. In this study, the ratio of *Bax/Bcl-2* was considered in order to determine the occurrence of apoptosis. According to the results shown in Figure 10, the treatment of MCF-7 cells with MPSE caused a significant decrease in the levels of *Bcl-2* and a notable increased level of *Bax* gene expression in a concentration-dependent manner. In addition, as the concentration increased, the *Bax/Bcl-2* ratio was significantly increased after MPSEs treatment in MCF-7 cells. Meanwhile, the expression levels of *PARP* were decreased slightly. Conclusively, the series of changes that occur in apoptosis-related gene expression values, along with the disruption of the mitochondrial membrane potential, indicate that MPSE could induce the apoptosis of MCF-7 cells via the intrinsic mitochondrial pathway.

## 4. Discussion

Today, many breast cancer treatments have failed to successfully cure patients because, in order to survive, cancer cells develop to avoid the process of apoptosis that is induced by conventional therapy protocols [6]. Thus, targeting apoptosis cell death in cancer cells can be part of a promising strategy in cancer therapies. Currently, plant-derived drugs, regardless of whether they are based on crude extracts or isolated bioactive compounds, have received much scientific attention with regard to cancer therapy due to their ability to modulate apoptosis. Moreover, they are relatively less toxic and present a lower probability of causing side effects [29, 30].

In this study, we reported that MPSE caused cytotoxic effects and inhibited the proliferation of breast cancer cells (MCF-7) in a dose- and time-dependent manner. Conversely, MPSE exerted less toxicity to immortalized normal breast cells (MCF-10A) with an IC_50_ value of more than 36.67 *μ*g/mL, suggesting that MPSE could be a promising anticancer agent in the treatment of cancer cells. Moreover, MPSE-induced G2/M phase arrested and triggered apoptosis signaling in MCF-7 breast cancer cell lines. Our findings contribute to a greater understanding of the strong positive relationship that exists between the phytochemical contents in MPSE and anticancer activities.

Phytochemical analysis of MPSE by HPLC and LC/MS analyses revealed the presence of a gallotannin-rich fraction in the extract that included pentagalloyl glucose (PGG). This was determined to be the main bioactive component in MPSE followed by ethyl gallate (EG) and gallic acid (GA), respectively. Previously, our report announced that the extraction yield of MPSE from ripe seeds of maprang (*Bouea macrophylla* Griffith) using 75% ethanol as extracted solvent was the highest value in maprang prieyo (11.92%) followed by maprang wan (11.72%) and mayongchid (10.08%), respectively [25]. The equations of Table 1 were used to standardize these three major compounds via HPLC analysis of three Thai varieties, and the results are presented in Table 4. The calculation of the total content of three compounds (%w/w; g of GA + EG + PGG/g of MPSE) has shown the highest value at 99.42% of maprang wan, follows by 80.48% of maprang prieyo and 45.92% of mayongchid, respectively. Estimation of the presence of three phytochemicals based on HPLC analysis at 100 kg of fresh ripe fruits was 1.03 kg PGG, 0.62 kg EG, and 0.05 kg GA for maprang wan, and was 0.77 kg PGG, 0.50 kg EG, and 0.04 kg GA for maprang prieyo. PGG (the main component in MPSE) has been shown to exert anticancer activity against both ER+ and ER– breast cancer cell lines. Additionally, PGG induced G0/G1 and S phase cell cycle arrest and apoptosis in T-47D and BT-474 cells by inhibiting cyclin D1 and by affecting specific apoptosis-related proteins such as Bax and Bcl-2 [31]. PGG was also capable of inhibiting triple-negative breast xenograft growth and metastasis via the inhibition of the JAK1-STAT3 pathway and by exerting antiangiogenesis, antiproliferation, and induced apoptosis [32]. Ethyl gallate (EG) was the second main bioactive compound in MPSE. EG has been demonstrated to suppress the proliferation and invasion of breast cancer cells. Suppression was modulated via the PI3K/Akt pathway, while treatment with EG decreased the activity of matrix metalloproteinase-2 (MMP-2) and MMP-9 in MDA-MB-231 cells. Induction of apoptosis was regulated by the altering of Bax/Bcl-2 ratio [33]. Another polyphenol compound, gallic acid (GA), was also found to display anticancer activity in MCF-7 cells. This compound has been demonstrated to induce antiproliferation activity and apoptosis in MCF-7 cells via increasing the levels of p27^Kip1^ and p21^Cip1^ and by decreasing the proliferation and induction of G2/M phase cell cycle arrest [34]. Based on the anticancer properties possessed by PGG, EG, and GA, which are the main components of the bioactive compounds in MPSE, it can be concluded that the antiproliferative effect of MPSE against MCF-7 cells may be due to the presence of these bioactive compounds or to their synergistic effects in the crude extract.

To explore the molecular mechanism underlying the anticancer activity of MPSE, cell cycle distribution, disruption of mitochondria membrane potential, apoptosis induction, and apoptosis-related genes were investigated. It is well known that cell cycle arrest in response to DNA damage or cellular stress is integral to the maintenance of genomic integrity. The cell cycle checkpoints play a crucial role in controlling the mechanisms that restrain cell cycle transition or in inducing apoptotic signaling pathways after cell stress [35]. The analysis of the cell cycle revealed that the MPSE-treated MCF-7 cells were arrested in the G0/G1 and G2/M phases of the cell cycle together with an increase in the sub-G1 population, which suggested the occurrence of the sequential events of cell cycle arrest that were followed by apoptosis. These results indicate that MPSE may act to inhibit cell proliferation via G2/M phase arrest in a dose-dependent manner. The G2 checkpoint was regulated by the activation of multiple pathways that act together to inhibit the activity of the cyclin B1/cdc2 kinase complex. Additionally, p53 and p21 appear to be essential for maintaining the cell cycle checkpoint in human cells. The upregulation of p53 and p21 upon cellular stress or DNA damage can cause cell cycle arrest at the G1, G2, or S phases via interaction with a wide range of cyclin/CDK complexes and is an important key in altering cancer growth [36]. Pentagalloyl glucose (PGG), which is mainly found in MPSE, is the precursor of gallotannin and has been shown to induce the cell cycle arrest and apoptosis [37]. Furthermore, a study by Chen et al. described the inhibitory effect of PGG in MCF-7 cells via the strong induction of G1 phase cell cycle arrest. PGG gradually increased the levels of p27 and p21, which were inhibitors of cyclin/CDK complexes in the G1-phase, resulting in the accumulation of G0/G1 phase cells after PGG treatment, and contributed to the anticancer activity of PGG [38]. Our preliminary study showed that MPSE induced cell cycle retardation in the G2/M phase by increasing the expression of p21 and by suppressing cyclin B1 and D1 (unpublished; manuscript in preparation). We also examined various apoptotic markers in MPSE-treated MCF-7 cells. After treatment with MPSE, the MCF-7 cells revealed condensed chromatin and apoptotic bodies (Figure 8(a)) and increased the percentage of apoptotic cells, as was confirmed by flow cytometric Annexin-V FITC analysis. Therefore, our results suggest that both apoptosis induction and G0/G1 and G2/M phase cell cycle arrest contributed to the anticancer activity of MPSE.

Apoptosis is a fundamental process that is essential for both the development and maintenance of tissue homeostasis. There are two principal signaling transduction pathways involved in the process of apoptosis: one is mitochondria-independent pathway and the other is the mitochondria-dependent pathway. The mitochondria-dependent pathway of apoptosis was triggered by DNA damage or an increase of ROS. It has been widely reported that ROS plays a crucial role in triggering cell damage, cell cycle progression, and cell death [39, 40]. In this study, we reported that MPSE treatment could affect intracellular ROS production and mitochondrial dysfunction in breast cancer cells. Base on the data obtained from the DCFH-DA assay, MPSE was found to increase the production of ROS in MCF-7 cells in a dose- and time-dependent manner. It started inducing ROS generation as early as 3 h and reached to its maximum value in 6 h, and then the ROS level tended to decrease gradually to the basal level in 12 h of treatment. This decrease of ROS may result from multiple adaptive mechanisms of cancer cells to protect the cells against oxidative stress [41]. When ROS rose to a certain level at the upper limit value of oxidative stress, the antioxidation systems were turned on to diminish an excess ROS and decrease the ROS level to the basal state. Thus, the apparent ROS levels of MPSE-induction cells were positively and negatively oscillated around the basal value which depends on the power of MPSE on ROS generation and the capability of antioxidant systems of those cells. However, treatment of MCF-7 cells at the high concentration of MPSE (60 *μ*g/mL) showed an increase ROS levels at 24 h of incubation, which was strongly linked to a decrease in ΔΨm (Figure 8). This higher oxidative stress could be explained by the enhanced mitochondrial rupture, considering that MPSE induced the excessive ROS damage to the cells *via* oxidizing lipids in mitochondrial membranes and the damaged cells produced more ROS [42]. As shown in the results (Figure 8), the MPSE at 60 *μ*g/mL treated MCF-7 cell line had a slight increase in green fluorescent of JC-1 monomer, an indicator of mitochondrial membrane rupture and disruption of mitochondrial membrane potential. However, there were fewer changes of the red/green fluorescent intensity ratio in MCF-7 treated with 30 *μ*g/mL MPSE. This suggested that, at the concentration of 30 *μ*g/mL MPSE, the ROS generation has no effect on the damage to the mitochondrial membrane. This result indicated that the MCF-7 breast cancer cell line treated with 60 *μ*g/mL MPSE had elevated levels of ROS generation at 24 h treatment, in part due to the increase in the release of ROS from the damaged cells, a process so-called ROS-induced ROS release (RIRR) [42]. However, a further in-depth study focusing on the mitochondrial ROS generation, determined by mitoSOX correlation with the effect of MPSE-treated breast cancer cells, must be performed to confirm this.

Moreover, ROS produced in the mitochondria has been linked to mitochondrial membrane rupture and the loss of mitochondrial membrane potential (ΔΨm). This outcome was subsequently associated with the mitochondrial release of the proapoptotic protein [43]. We observed the increased levels of ROS after MPSE treatment, as well as MPP disruption in treated MCF-7 cells. The MPSE treatment collapsed ΔΨm in MCF-7 cells in a dose-dependent manner. These results indicated that an increase in intracellular ROS in breast cancer cells may be a result of a decrease in ΔΨm. It would then act to trigger apoptosis through the mitochondria pathway. A previous study also reported similar results in which the aglycone-rich extract of phytoestrogens could induce an increase of ROS, which could then modulate the mitochondrial membrane potential and trigger and release certain mitochondrial proapoptotic factors such as cytochrome c, all of which would ultimately activate the mitochondria-mediated apoptosis pathway [13, 44].

Mitochondria-mediated apoptosis is regulated by antiapoptotic proteins such as Bcl-2 and proapoptotic proteins such as Bax. Subsequently, the Bax/Bcl-2 ratio appears to be a critical determinant for cell survival or cell death [45]. Our results revealed that the MPSE treatment could result in the upregulation of *Bax* and the downregulation of *Bcl-2* mRNA expression which would then trigger the progression of apoptosis. Therefore, we have inferred that the change in the ratio of pro- and antiapoptotic genes might largely contribute to the mitochondria-mediated apoptosis pathway. Similar results have been reported by Mun et al., wherein they found that *Galla Rhois* extract contains several significant components such as methyl gallate, gallic acid, PGG, and gallotannin, all of which act to suppress the viability of colorectal cells by inducing apoptosis through the cleavage of caspase-3 and PARP; the downregulation of caspase-8, caspase-9, Bcl-2, and Bcl-xL; and the upregulation of Bax [46]. In addition, some chemical agents have been reported for their anti-breast cancer activities such as oxymatrine. The effects of oxymatrine in human breast cancer MCF-7 cells using real-time PCR and Western blot analysis had been investigated. The results found that oxymatrine enhanced the expression of Bax protein while reducing the expression of Bcl-2 protein. Oxymatrine treatment showed proapoptotic effects in breast cancer MCF-7 cells, and these effects correlated with the upregulation of *Bax* transcription and protein expression and the downregulation of *Bcl-2* transcription and protein expression in a time- and dose-dependent manner. This finding revealed that the oxymatrine had effects in promoting apoptosis in human breast cancer MCF-7 cells by mediating the mRNA and protein expression levels of Bax and Bcl-2 [47].

## 5. Conclusion

In conclusion, the results of our study suggest that the main MPSE constituents, PGG, EG, and GA, induced oxidative stress in treated MCF-7 cells. This outcome has led to the loss of mitochondrial membrane permeability and to an increased ratio of *Bax/Bcl-2* gene expression, which activated the mitochondria-mediated apoptosis pathway. Moreover, MPSE also induced G2/M phase cell cycle arrest resulting in the suppression of MCF-7 cell proliferation. Our results indicate that gallotannin-rich extracts obtained from maprang seeds might be potentially used in the development of an alternative drug that could target the apoptosis pathway in breast cancer cells.

## Figures and Tables

**Table 1:** Limit of detection (LOD) and limit of quantification (LOQ) of GA, EG, and PGG of maprang seed extract.

	Linear equation *Y* = *m* *∗* *X* + *c*	*R*-square (COD)	SD (*n* = 7)	LOD (*μ*g/mL) 3.3 SD/slope	LOQ (*μ*g/mL) 10 SD/slope
Gallic acid (GA)					
Area peak at 271 nm	*Y* = 59579.31 *∗* *X* − 41513.36	0.99918	2022.81	0.11	0.34
Area peak at 279 nm	*Y* = 52126.87 *∗* *X* − 29251.57	0.99924	1698.69	0.11	0.32

Ethyl gallate (EG)					
Area peak at 271 nm	*Y* = 35918.44 *∗* *X* + 151845.02	0.99953	925.89	0.08	0.26
Area peak at 279 nm	*Y* = 32101.91 *∗* *X* + 151521.91	0.99949	858.25	0.09	0.27

Pentagalloyl glucose (PGG)					
Area peak at 271 nm	*Y* = 37788.23 *∗* *X* − 430.14	0.99945	1044.24	0.09	0.28
Area peak at 279 nm	*Y* = 41639.62 *∗* *X* + 1730.32	0.99946	1144.46	0.09	0.27

**Table 2:** m/z ratio of the three major phytochemicals of maprang seed extract using LC-MS analysis with API-ES mode.

Rt (min)	m/z	[M-H]^+^	Identification	Product ions
4.764	171.1	[GA-H]^+^	Gallic acid	194.1, 171.1, 144.1/145.1, 127.1, 101.2

22.501	199.1	[EG-H]^+^	Ethyl gallate	221.1/222.1, 199.1/200.1, 171.1
	221.1	[EG-Na]^+^		

28.670	959.0	[PGG.H_2_O-H]^+^	Pentagalloyl glucose	980.0/981.0, 963.1/964.1, 958.0/959.0, 771.0/772.1/773.1, 185.1, 180.2/181.9, 164.1/163.1
	963.1	[PGG-Na]^+^		
	980.0	[PGG-K]^+^		
	981.0	[PGG.H_2_O-Na]^+^		

**Table 3:** Cytotoxicity of the compounds isolated from maprang seed extracts towards MCF-7, MCF-10A, and MDA-MB-231 cells at 72 h as reflected by IC_50_ values as determined by MTT assay.

Compounds	MCF-7 IC_50_ (*μ*g/mL)	MCF-10A IC_50_ (*μ*g/mL)	MDA-MB-231 IC_50_ (*μ*g/mL)
MPSE	6.94 ± 1.57	30.08 ± 3.70	18.92 ± 1.20
PGG	>100	>100	26.46 ± 6.53
EG	48.16 ± 0.56	>100	18.04 ± 1.50
GA	26.23 ± 0.24	26.23 ± 0.24	7.39 ± 1.51

Data are represented as IC_50_ ± SD of three replicates from three independent tests.

**Table 4:** Abundance of the three major phytochemicals, GA, EG, and PGG, presented in the maprang seed kernels, ripe fruits of 3 Thai maprang varieties, maprang wan, maprang prieyo, and mayongchid.

Maprang variety	MPSE extraction yield^*∗*^		Abundance (%w/w)											
	kg MPSE/100 kg FS	kg MPSE/100 kg FF	GA			EG			PGG			GA + EG + PGG		
			MPSE	FS	FF	MPSE	FS	FF	MPSE	FS	FF	MPSE	FS	FF
Maprang wan	11.72	1.72	3.09	0.36	0.05	36.20	4.24	0.62	36.20	7.05	1.03	99.42	11.65	1.71
Maprang prieyo	11.92	1.62	2.45	0.29	0.04	30.68	3.66	0.50	30.68	5.64	0.77	80.48	9.59	1.30
Mayongchid	10.08	0.57	3.10	0.31	0.02	17.68	1.78	0.10	17.68	2.53	0.14	45.92	4.63	0.26

The standardization of GA, EG, and PGG by HPLC analysis. MPSE was determined at 1,000 *μ*g/mL (*n* = 3). FF: fresh fruit; FS: fresh seed. ^*∗*^Data were obtained from [25].

## Data Availability

The data used to support the findings of this study are available from the corresponding author upon request.

## References

[1] Mitra S., Dash R. (2018). Natural products for the management and prevention of breast cancer. *Evidence-Based Complementary and Alternative Medicine*.

[2] Geng L., Wang J. (2017). Molecular effectors of radiation resistance in colorectal cancer. *Precision Radiation Oncology*.

[3] Zhang M., Liu E., Cui Y., Huang Y. (2017). Nanotechnology-based combination therapy for overcoming multidrug-resistant cancer. *Cancer Biology & Medicine*.

[4] Singh S., Sharma B., Kanwar S. S., Kumar A. (2016). Lead phytochemicals for anticancer drug development. *Frontier Plant Science*.

[5] Lauber K., Ernst A., Orth M., Herrmann M., Belka C. (2012). Dying cell clearance and its impact on the outcome of tumor radiotherapy. *Frontier Oncology*.

[6] Tor Y. S., Yazan L. S., Foo J. B. (2015). Induction of apoptosis in MCF-7 cells via oxidative stress generation, mitochondria-dependent and caspase-independent pathway by ethyl acetate extract of *Dillenia suffruticosa* and its chemical profile. *PLoS One*.

[7] Brenner D., Mak T. W. (2009). Mitochondrial cell death effectors. *Current Opinion in Cell Biology*.

[8] Rampal G., Khanna N., Thind T. S., Arora S., Vig A. P. (2012). Role of isothiocyanates as anticancer agents and their contributing molecular and cellular mechanisms. *Medicinal Chemistry & Drug Discovery*.

[9] Zong W.-X., Rabinowitz J. D., White E. (2016). Mitochondria and cancer. *Molecular Cell*.

[10] Moloney J. N., Cotter T. G. (2018). ROS signalling in the biology of cancer. *Seminars in Cell & Developmental Biology*.

[11] Seca A., Pinto D. (2018). Plant secondary metabolites as anticancer agents: successes in clinical trials and therapeutic application. *International Journal of Molecular Sciences*.

[12] Mileo A. M., Miccadei S. (2016). Polyphenols as modulator of oxidative stress in cancer disease: new therapeutic strategies. *Oxidative Medicine and Cellular Longevity*.

[13] Kavitha N., Ein Oon C., Chen Y., Kanwar J. R., Sasidharan S. (2017). *Phaleria macrocarpa* (Boerl.) fruit induce G_0_/G_1_ and G_2_/M cell cycle arrest and apoptosis through mitochondria-mediated pathway in MDA-MB-231 human breast cancer cell. *Journal of Ethnopharmacology*.

[14] Torres-León C., Ventura-Sobrevilla J., Serna-Cock L., Ascacio-Valdés J. A., Contreras Esquivel J. C. (2017). A valuable phenolic compound with functional properties. *Journal of Function Foods*.

[15] Zhang J., Li L., Kim S. H., Hagerman A. E., Lü J. (2009). Anti-cancer, anti-diabetic and other pharmacologic and biological activities of penta-galloyl-glucose. *Pharmaceutical Research*.

[16] Lin V. C.-H., Kuo P.-T., Lin Y.-C. (2014). Penta-O-galloyl-β-d-glucose suppresses EGF-induced eIF3i expression through inhibition of the PI3K/AKT/mTOR pathway in prostate cancer cells. *Journal of Agricultural and Food Chemistry*.

[17] Kant R., Yen C. H., Lu C. K., Lin Y. C., Li J. H., Chen Y. M. (2016). Identification of 1,2,3,4,6-penta-O-galloyl-β-d-glucopyranoside as a glycine N-methyltransferase enhancer by high-throughput screening of natural products inhibits hepatocellular carcinoma. *International Journal of Molecular Sciences*.

[18] Huang C., Lee S.-Y., Lin C.-L. (2013). Co-treatment with quercetin and 1,2,3,4,6-penta-O-galloyl-β-d-glucose causes cell cycle arrest and apoptosis in human breast cancer MDA-MB-231 and AU565 cells. *Journal of Agricultural and Food Chemistry*.

[19] Nithitanakool S., Pithayanukul P., Bavovada R. (2009). Antioxidant and hepatoprotective activities of Thai mango seed kernel extract. *Planta Medica*.

[20] Kiss A. K., Naruszewicz M. (2012). Polyphenolic compounds characterization and reactive nitrogen species scavenging capacity of Oenothera paradoxa defatted seed extracts. *Food Chemistry*.

[21] Cargnin S. T., Gnoatto S. B. (2017). Ursolic acid from apple pomace and traditional plants: a valuable triterpenoid with functional properties. *Food Chemistry*.

[22] Babbar N., Oberoi H. S., Sandhu S. K. (2015). Therapeutic and nutraceutical potential of bioactive compounds extracted from fruit residues. *Critical Reviews in Food Science and Nutrition*.

[23] Sagar N. A., Pareek S., Sharma S., Yahia E. M., Lobo M. G. (2018). Fruit and vegetable waste: bioactive compounds, their extraction, and possible utilization. *Comprehensive Reviews in Food Science and Food Safety*.

[24] Suttana W., Dechsupa N., Mankhetkorn S. (2013). Preparation of maprang seed extracts and evaluation their anti-proliferative activity against drug-sensitive and drug-resistant leukemia and lung cancer cells. *Srinagarind Medical Journal*.

[25] Nathupakorn D., Kantapan J., Tungjai M., Intorasoot S. (2019). Maprang “*Bouea macrophylla* Griffith” seeds: proximate composition, HPLC fingerprint, and antioxidation, anticancer and antimicrobial properties of ethanolic seed extracts. *Heliyon*.

[26] Paksee S., Kantapan J., Chawapun P., Sangthong P., Dechsupa N. (2019). Maprang seed extracts suppressed chemoresistant properties of breast cancer cells survived from ionizing radiation treatments via the regulation of ABCB1 Genes. *International Journal of Medical Sciences*.

[27] Paksee S. (2019). Phenotypic investigation of minimal residual breast cancer cell lines from Maprang seed extracts combined with X-ray irradiation.

[28] International Committee on Harmonization (2019). ICH harmonised tripartite guidelines: validation of analytical procedures: text and methodology.

[29] Fulda S. (2010). Modulation of apoptosis by natural products for cancer therapy. *Planta Medica*.

[30] Wang Y., Zhong J., Bai J. (2018). The application of natural products in cancer therapy by targeting apoptosis pathways. *Current Drug Metabolism*.

[31] Xiang Q., Tang J., Luo Q. (2019). In vitro study of anti-ER positive breast cancer effect and mechanism of 1,2,3,4-6-pentyl-O-galloyl-beta-D-glucose (PGG). *Biomedicine & Pharmacotherapy*.

[32] Lee H.-J., Seo N.-J., Jeong S.-J. (2011). Oral administration of penta-O-galloyl- -D-glucose suppresses triple-negative breast cancer xenograft growth and metastasis in strong association with JAK1-STAT3 inhibition. *Carcinogenesis*.

[33] Cui H., Yuan J., Du X., Wang M., Yue L., Liu J. (2015). Ethyl gallate suppresses proliferation and invasion in human breast cancer cells via Akt-NF-κB signaling. *Oncology Reports*.

[34] Hsu J.-D., Kao S.-H., Ou T.-T., Chen Y.-J., Li Y.-J., Wang C.-J. (2011). Gallic acid induces G2/M phase Arrest of breast cancer cell MCF-7 through stabilization of p27Kip1Attributed to disruption of p27Kip1/skp2 complex. *Journal of Agricultural and Food Chemistry*.

[35] Flatt P. M., Pietenpol J. A. (2000). Mechanisms of cell-cycle checkpoints: at the crossroads of carcinogenesis and drug discovery. *Drug Metabolism Reviews*.

[36] Georgakilas A. G., Martin O. A., Bonner W. M. (2017). p21: a two-faced genome guardian. *Trends in Molecular Medicine*.

[37] Kwon T.-R., Jeong S.-J., Lee H.-J. (2012). Reactive oxygen species-mediated activation of JNK and down-regulation of DAXX are critically involved in penta-O-galloyl-beta-D-glucose-induced apoptosis in chronic myeloid leukemia K562 cells. *Biochemical and Biophysical Research Communications*.

[38] Chen W.-J., Chang C.-Y., Lin J.-K. (2003). Induction of G1 phase arrest in MCF human breast cancer cells by pentagalloylglucose through the down-regulation of CDK4 and CDK2 activities and up-regulation of the CDK inhibitors p27Kip and p21Cip. *Biochemical Pharmacology*.

[39] Redza-Dutordoir M., Averill-Bates D. A. (2016). Activation of apoptosis signalling pathways by reactive oxygen species. *Biochimica et Biophysica Acta (BBA)—Molecular Cell Research*.

[40] Carrasco-Torres G., Monroy-Ramírez H. C., Martínez-Guerra A. A. (2017). Quercetin reverses rat liver preneoplastic lesions induced by chemical carcinogenesis. *Oxidative Medicine and Cellular Longevity*.

[41] Lara G., Marcello P., Milena N. (2010). Interfering with ROS metabolism in cancer cells: the potential role of quercetin. *Cancers*.

[42] Park J., Lee J., Choi C. (2011). Mitochondrial network determines intracellular ROS dynamics and sensitivity to oxidative stress through switching inter-mitochondrial messengers. *PLoS One*.

[43] Xiao D., Powolny A. A., Singh S. V. (2008). Benzyl isothiocyanate targets mitochondrial respiratory chain to trigger reactive oxygen species-dependent apoptosis in human breast cancer cells. *Journal of Biological Chemistry*.

[44] Dutta S., Khanna A. (2016). Aglycone rich extracts of phytoestrogens cause ROS-mediated DNA damage in breast carcinoma cells. *Biomedicine & Pharmacotherapy*.

[45] Chen X.-X., Leung G. P.-H., Zhang Z.-J. (2017). Proanthocyanidins from *Uncaria rhynchophylla* induced apoptosis in MDA-MB-231 breast cancer cells while enhancing cytotoxic effects of 5-fluorouracil. *Food and Chemical Toxicology*.

[46] Mun J. G., Kee J. Y., Han Y. H. (2018). Galla Rhois water extract inhibits lung metastasis by inducing AMPK-mediated apoptosis and suppressing metastatic properties of colorectal cancer cells. *Oncology Reports*.

[47] Lin B., Li D., Zhang L. (2016). Oxymatrine mediates Bax and Bcl-2 expression in human breast cancer MCF-7 cells. *Pharmazie*.

